# Predictive Factors for Gastrointestinal and Genitourinary Toxicities in Prostate Cancer External Beam Radiotherapy: A Scoping Review

**DOI:** 10.3390/diagnostics15111331

**Published:** 2025-05-26

**Authors:** Jerry C. F. Ching, Kelvin C. K. Liu, Isaac K. H. Pang, Alexander J. Nicol, Vincent W. S. Leung, Jing Cai, Shara W. Y. Lee

**Affiliations:** 1 Department of Health Technology and Informatics, The Hong Kong Polytechnic University, Hong Kong, China; jerrycf.ching@connect.polyu.hk (J.C.F.C.); chun-kit-kelvin.liu@connect.polyu.hk (K.C.K.L.); ka-him.pang@connect.polyu.hk (I.K.H.P.); alexander.nicol@connect.polyu.hk (A.J.N.); wsv.leung@polyu.edu.hk (V.W.S.L.); 2 Shenzhen Research Institute, The Hong Kong Polytechnic University, Shenzhen 518000, China; 3 Research Institute for Intelligent Wearable Systems, The Hong Kong Polytechnic University, Hong Kong, China

**Keywords:** prostate cancer, genitourinary toxicity, gastrointestinal toxicity, acute toxicity, late toxicity, predictive factors

## Abstract

Advancements in radiotherapy (RT) techniques such as intensity modulation, image guidance, and hypofractionation have facilitated a satisfactory survival outcome in prostate cancer (PCa) patients. However, virtually all PCa patients suffer from various types and extents of radiation toxicities, which are mainly gastrointestinal (GI) and genitourinary (GU) in nature, eroding their quality of life. Thus, early mitigation and preventative measures should be offered, enabled by accurate toxicity prediction. This scoping review provides a comprehensive summary of reported acute and late GI and GU toxicity predictors of conventional fractionation (CFRT), moderate hypofractionation (MHRT), and ultra-hypofractionation (UHRT). A total of 169 studies published between the years 2000 and 2024 (inclusive) were identified from four databases, with 127 and 78 studies investigating GI and GU toxicities, respectively. Univariate analysis was employed in 139 studies to identify predictors, while 94 studies involved multivariate analysis, 40 involved internal model validation, and 5 performed external model validation. Among all studies, dosimetric predictors are the most reported factors, followed by patient, clinical, treatment, disease, genetic, and radiomic features. However, their applicability and performance have not yet been extensively proven in external validation involving multicenter studies. Future predictive studies should also focus on deeper multimodality information, such as radiomics, in addition to the categories of factors consolidated in this study, for an all-rounded investigation. A multicenter study is highly encouraged for prospective external validation. Further investigations into delivered doses and sub-volumes of various regions of interest are necessary. Comprehensive reporting items suggested in this work shall facilitate the reproducibility and comparability of the results.

## 1. Introduction

Prostate cancer (PCa) is one of the most diagnosed cancers among men globally, accounting for approximately 1.5 million new cases and 375,000 deaths annually [1]. Incidence is projected to reach 2.9 million in 2040 [2]. Currently, it is also the most common male cancer diagnosed in over half of countries worldwide [3].

Localized PCa is traditionally stratified into low, intermediate, and high-risk groups based on clinical stage, Gleason score, and PSA [4]. Several international guidelines exist [5,6]. The National Comprehensive Cancer Network (NCCN) also subdivides intermediate risk into favorable/unfavorable and includes a very-high-risk category [7]. Patients with low-risk or indolent disease often choose watchful waiting or active surveillance [4]. External beam radiotherapy (EBRT) is widely used across risk levels, with radiotherapy (RT) advancing from three-dimensional radiotherapy (3DCRT) to intensity-modulated radiotherapy (IMRT) aided by image-guided radiotherapy (IGRT) [8,9,10], improving dose conformity and minimizing gastrointestinal (GI) and genitourinary (GU) toxicities [11,12]. Because the alpha–beta ratio of prostate adenocarcinoma is low (0.47–4.14), higher biologically equivalent doses can be delivered via hypofractionation [13]. Consequently, PCa RT fractionation has shifted from conventional (CFRT) to moderate (MHRT) or ultra-hypofractionated (UHRT).

It is evident that both CFRT and MHRT yield satisfactory disease control, both attaining 5-year disease-free survival (DFS) of above 85% in a recent meta-analysis of phase 3 randomized controlled trials involving low to high-risk PCa patients [14]. While the current NCCN has not yet recommended UHRT in high-risk patients, its performance in disease control has been satisfactory in a meta-analysis, achieving 5-year biochemical failure-free survival (bFFS) of over 92% in both low and intermediate-risk patients [15]. The latest phase 3 trial of UHRT (PACE-B) found the 5-year incidence of freedom from biochemical or clinical failure to be 95.8% and is non-inferior to CFRT [16]. With a satisfactory survival period, radiation toxicity management has been of equivalent importance to disease control, if not higher [17].

Grading systems have been developed to standardize the assessment and reporting of treatment toxicities. The Common Terminology Criteria for Adverse Events (CTCAE) [18] and the Radiation Therapy Oncology Group (RTOG) criteria [19] are widely used clinician-reported outcome (CRO) scales for grading the severity of treatment-related toxicities. These systems typically range from grade 1 (mild) to grade 5 (death), with higher grades indicating more severe symptoms. Of note, RTOG adopts overall grading for a type of toxicity while CTCAE provides individual grading for each symptom. Additionally, patient-reported outcome measures such as the International Prostate Symptom Score (IPSS) [20] are often employed to capture the patient’s perspective on urinary symptoms and quality of life. GU and GI toxicities are major side effects of PCa radiotherapy, significantly impacting patients’ quality of life [21,22].

GI toxicities primarily affect the rectum and anal canal. Approximately 10% to 50% of patients treated by CFRT or HFRT experience moderate to severe acute GI side effects, including proctitis, diarrhea, and abdominal pain, which can affect quality of life during and after treatment [23]. Meta-analysis estimates a summary effect size of 12.1% and 14.6% incidence of late grade 2+ GI toxicities [24]. Assessment may involve endoscopic evaluation in addition to patient-reported symptoms. It is emphasized that even late fecal incontinence occurs in only about 5% of patients, and it strongly erodes quality of life [25]. Proposed mitigation strategies include refining dose constraints for organ-at-risk (OAR), using IMRT or volumetric modulated arc therapy (VMAT) techniques, and implementing rectal spacers to increase the distance between the prostate and rectum [26]. Additionally, systematic review and meta-analysis suggest the use of probiotics and synbiotics for the mitigation of GI toxicities [27]. Nevertheless, careful patient selection and adherence to dose constraints remain crucial in hypofractionation [28,29].

GU toxicities typically manifest as urinary frequency, urgency, incontinence, retention, dysuria, and hematuria. Meta-analysis estimates a summary effect size of 19.4% and 20.4% incidence of late grade 2+ GU toxicities, in CFRT and MHRT cohorts, respectively [14]. Up to 34% of patients treated by UHRT may experience acute GU toxicities [30]. Current mitigation strategies include optimizing treatment planning to reduce the dose to the bladder and urethra, using IMRT, and exploring the potential of adaptive radiotherapy based on the accumulated dose [31,32].

Furthermore, GI and GU toxicities may increase upon hypofractionation. A meta-analysis reveals an increase in acute Grade 2+ GI and GU toxicities [24]. A phase 3 randomized trial comparing CFRT and MHRT also revealed a heightened rate of late Grade 2+ toxicities at three years after radiotherapy, violating the non-inferiority criteria [33]. Late toxicity incidence in UHRT is also significantly higher than that of CFRT or MHRT in the PACE-B trial [21]. Preliminary results from PACE-C also show an increasing trend of both acute GI and GU toxicities under the CTCAE scale in the UHRT cohort.

In view of the importance of PCa patient QoL, accurate prediction of toxicities and patient selection are prerequisites for the timely implementation of preventative or mitigative measures. Previous systematic reviews have either investigated predictors from one type of fractionation scheme or combinedly analyzed any two types of fractionation schemes [34,35]. It is well agreed that normal tissues often respond in different periods under CFRT, MHRT, or UHRT. In addition, different RT techniques are often required for various fractionation schemes, such as image guidance, patient positioning tolerance, and planning constraints. Thus, this scoping review aims to perform a systematic, broad search and consolidate GI and GU toxicities predictive factors among PCa patients treated by various fractionation schemes. Synthesized knowledge should be considered in future modelling studies for clinical use.

## 2. Materials and Methods

**Figure 1:**
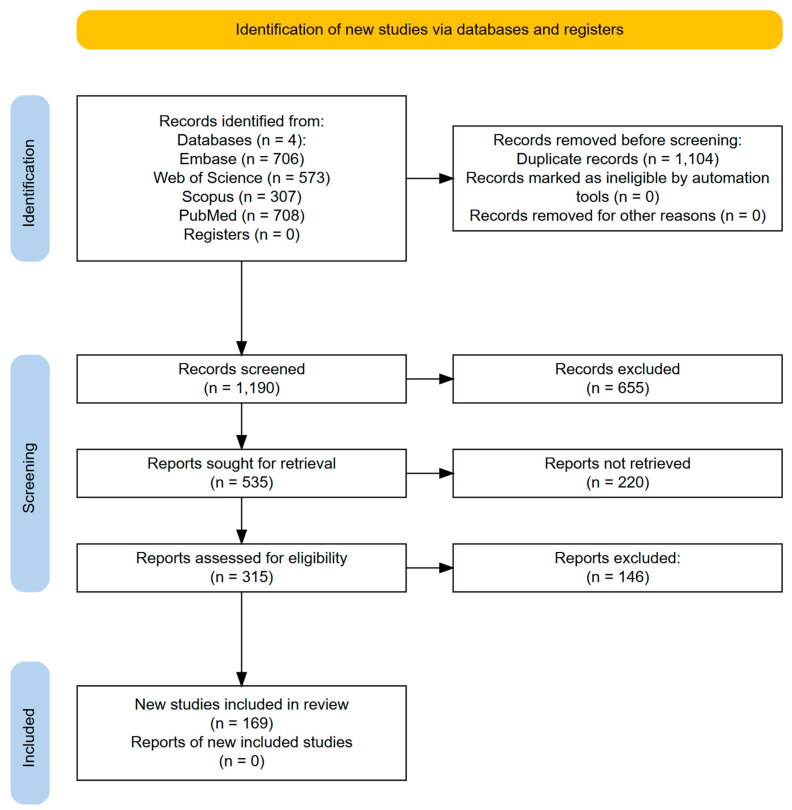
PRISMA flow diagram.

A systematic literature search was conducted according to the *Preferred Reporting Items for Systematic Review and Meta-Analysis Protocols Extension for Scoping Reviews* (PRISMA-ScR) guidelines [36,37]. The PRISMA-ScR checklist is available in the Supplementary Materials. The primary aim was to identify studies that report predictive factors for GI and/or GU toxicities in PCa patients treated by CFRT, MHRT, or UHRT. The secondary aim was to report on the relevant machine learning (ML) or artificial neural network (ANN)-based predictive models. Searches were conducted on Embase, Web of Science, Scopus, and PubMed databases on 31 December 2024. The search strategy for each database is listed in Table A1. The flow diagram for the selection of sources of evidence is displayed in Figure 1.

### 2.1. Inclusion and Exclusion Criteria

Literature was included if all the following inclusion criteria were met:

1. Published in 2000 or after;
2. Investigating primary PCa;
3. Using photon EBRT as primary treatment.

Literature was excluded if any of the following exclusion criteria were met:

1. Previous prostatectomy;
2. Salvage radiotherapy;
3. Brachytherapy involved;
4. Radiotherapy for recurrent PCa or re-irradiation;
5. Particle or non-photon radiation therapy;
6. Two-dimensional dosimetric planning;
7. No toxicity predictors provided;
8. Non-experimental study (including but not limited to reviews, opinions, letters, abstract or book chapters);
9. Full text unavailable;
10. Full text not in English.

### 2.2. Data Extraction

Phase one screening was performed on the title and abstract after duplicate removal. Full-text publications were screened for eligibility in phase two. Quality assessment was not performed on included publications for further evaluation or exclusion, as the current work sought to provide an overview of any toxicity research performed on the concerned cohort of CHRT, MHRT, and UHRT PCa patients [36,37,38]. Hence, as much of the relevant literature as possible is involved, with the aim of providing valuable insights for modelling studies in the future. It should also be noted that the highly heterogeneous nature and quantity of included studies render such an assessment impractical in a timely manner [38].

After determining the final set of publications to be included, data charting was performed to systematically extract details from each publication. Major extracted attributes include sample size, prostate risk level, primary treatment region-of-interest, side-effect scale, radiotherapy technique, dose scheme, and toxicity predictors. Particularly, toxicity predictors were specified as significant in univariate and/or multivariate analysis, and whether it was included in an externally validated model. After data extraction, publications were sorted according to the toxicity timeframe (acute and late), toxicity nature (GI and GU), and fractionation (CFRT, MHRT, and UHRT). The frequency of a predictor being reported as significant was defined as the amount of its supporting evidence [38]. Predictors were categorized by their nature and ranked by occurrence frequency based on the retrieved results. Publications involving ML or ANN models are arranged in another table. 

## 3. Results

### 3.1. Overview of Included Studies

The literature search identified 1190 unique records from four databases, of which 655 were excluded after phase one screening (Figure 1). Table 1 presents a summary of the 169 full-text articles included in this review [17,28,29,31,32,39,40,41,42,43,44,45,46,47,48,49,50,51,52,53,54,55,56,57,58,59,60,61,62,63,64,65,66,67,68,69,70,71,72,73,74,75,76,77,78,79,80,81,82,83,84,85,86,87,88,89,90,91,92,93,94,95,96,97,98,99,100,101,102,103,104,105,106,107,108,109,110,111,112,113,114,115,116,117,118,119,120,121,122,123,124,125,126,127,128,129,130,131,132,133,134,135,136,137,138,139,140,141,142,143,144,145,146,147,148,149,150,151,152,153,154,155,156,157,158,159,160,161,162,163,164,165,166,167,168,169,170,171,172,173,174,175,176,177,178,179,180,181,182,183,184,185,186,187,188,189,190,191,192,193,194,195,196,197,198,199,200,201,202], with 127 (75.1%) reporting GI toxicities and 78 (46.2%) reporting GU toxicities. A detailed distribution of studies on various toxicity endpoints and gradings is provided in the Supplementary Materials (Tables S1–S5). The average incidences of grade 1+ acute GI and GU toxicities are 48.3% and 53.5%, while those of late GI and GU toxicities are 23.3% and 37.2%, respectively. The average incidences of grade 2+ acute GI and GU toxicities are 19.3% and 28%, while those of late GI and GU toxicities are 14.9% and 16.1%, respectively. The median patient cohort size (n) was similar across both toxicities, with an overall median of 168, ranging from 9 to 3243 patients. Both the RTOG and the CTCAE scales were commonly used for toxicity grading, with the same adoption rates at 40.2%. The usage of versions two to five of CTCAE was reported. Due to historical effects, CFRT was the predominant treatment approach, accounting for 72.8% of studies, while MHRT and UHRT were implemented in 20.1% and 12.4% of studies, respectively. The availability of statistical analyses varied, with 82.2% of studies reporting univariate analysis and 55.6% conducting multivariate analysis. Despite the increasing emphasis on predictive modeling, only 23.7% of studies performed internal validation, and external validation was rare (3%).

Table 2, Table 3, Table 4 and Table 5 present the full distribution of predictors identified for acute and late GI and GU toxicities. Detailed distribution of predictors regarding each fractionation scheme (i.e., CFRT, MHRT, and UHRT) can be found in Supplementary Materials, Tables S6–S17. Each table consists of the specific toxicity outcome with or without grading specified, the predictor category, the predictor, and the number of articles with univariate and/or multivariate analysis supporting the predictor’s statistical significance. It is highlighted that multivariate analysis also accounts for interactions between multiple variables, but not in univariate analysis. A total of seven categories of predictors were identified: dosimetric, patient, clinical, treatment, disease, genetic, and radiomic features, in descending order of occurrence frequency. Dosimetric factors often refer to radiation dose parameters of the rectum, bladder, urethra, prostate gland, and their subregions. Conventional notations of V*x* or D*x* are defined as the volume receiving at least a dose of *x* Gy, or the highest dose received by *x* cc/% of tissue, respectively. Patient factors refer to individual patient characteristics or demographics, such as age, diabetes, drinking and smoking habits, and baseline urinary function. Clinical factors refer to pre-existing conditions or treatments, such as the use of anti-hypertensives, anticoagulants, prior abdominal surgery, and previous transurethral resection of the prostate (TURP). Disease factors involve the characteristics of the underlying disease, including prostate volume, clinical staging, and tumor risk group. Treatment-related factors include RT techniques such as IMRT and 3DCRT, and androgen deprivation therapy (ADT) regimens that may influence toxicity risk. Genetic factors refer to genetic predisposition, such as microRNA-related single-nucleotide polymorphisms (mirSNP), which may contribute to an increased radiation toxicity risk [78,153,154,177].

### 3.2. Predictors of Gastrointestinal Toxicities

#### 3.2.1. Acute Gastrointestinal Toxicities

In the context of CFRT, 16 clinical endpoints were identified from the literature (Table 2). Rectal dose consistently emerged as a key dosimetric predictor for acute GI toxicities, with reported associations spanning a broad dose range (V10–73) for G1+ toxicity [144,159] and more focused intervals (V37–70, Dmean) for G2 toxicity [49,88]. Specific subregions and dose regions (V65, V70, D2cc) were also implicated in G2+ GI toxicity [65,161,174], alongside structural geometry factors, such as cross-sectional area and surface area of the rectum [54,59,68,137]. Among patient factors, hemorrhoids [54,61,71,144] were the most frequently reported. Other patient factors including age [139], rectal volume [139], GI comorbidities [144], alcohol consumption [144], microbial alpha diversity [139], and history of diabetes mellitus [54] were frequently reported, while use of anti-coagulants [54] was linked to both G2+ toxicity and bleeding. Several clinical parameters, including previous abdominal/pelvic surgery [144] and TURP [139], appeared in G1+ GI toxicity, whereas pelvic nodal irradiation [54] emerged for G1+ and G2+ rectal endpoints. Hormone therapy or androgen deprivation [54,91] was associated with acute rectal toxicity. A set of biomarkers (pro-hepcidin, IL-6, TNF, hemoglobin, ferritin, transferrin) and genetic polymorphisms were linked to proctitis [55]. Specific symptom endpoints—such as rectal bleeding [54,61,71], diarrhea [42,54], incontinence [71], rectal urgency [54,71], tenesmus [54], stool frequency [54], and painful bowel movements [54,71], were likewise associated with rectal dose metrics, comorbidities (e.g., hemorrhoids), or treatment factors (e.g., irradiation of seminal vesicles).

The MHRT cohort was less studied for acute GI toxicities (Table 2). Among the included studies, the dosimetric factor still dominated the predictor set. Hot spots represented by Dmax or high dose region (V50–65) were predictive of G1–2 or the above acute GI toxicities [110,146]. Notably, the rectal wall alone was also associated with acute G2+ rectal bleeding [107]. A study found that high-dose amifostine, a cytoprotective adjuvant for kidney protection under chemotherapy, was associated with proctitis [77]. Meanwhile, statin medication was associated with acute G2+ GI toxicity [29].

The UHRT cohort was the least studied for acute GI toxicities, with only three studies (Table 2). Nevertheless, rectum V28 was linked with G1–2 GI toxicity [112] while V10–30, D50, Dmean, D25.3, and D10cc were associated with acute G2+ GI toxicity [186,192].

#### 3.2.2. Late Gastrointestinal Toxicities

Late GI toxicities in the CFRT context were investigated by the largest volume of studies, with 25 clinical endpoints identified (Table 3). The dosimetric factor was the most consistently identified predictor category of late GI toxicities. Moderate to high dose rectal regions of V35–70 predicted late G1+ GI toxicity [31,96,162]; rectum or rectal subregion V45–70, Dmean, D0.03cc, and D50% predicted more severe G2+ events. G1+ rectal bleeding was reportedly predicted by the whole-organ or subregion of the rectum or anorectal volume in nine studies [40,48,66,71,114,132,152,155,164]. Similar dose metrics have been reported in a series of studies to predict G2+ rectal bleeding (rectum or rectal subregion V30–75, Dmean, Dmax, EUD) [39,58,68,105,120,124,125,143,152,164,165], fecal incontinence (rectum V15–75) [58,61,105,109,164], stool frequency (rectum V15–75) [61,130,164], tenesmus (rectum or rectal subregion V50–65) [147,164], proctitis (rectum or rectal subregion V50–70) [66,155,168], rectal urgency (V50–75) [147,164], and abdominal pain [71,164]. Patient-specific factors frequently reported include age, consistently linked to G2+ GI toxicity [100], G3+ GI toxicity [100], and stool frequency [48]. Acute GI toxicity symptoms are significantly associated with numerous late toxicities, such as G1+, G2+, and G3+ GI toxicity [79,96,101,119,185,191]; G2+ rectal toxicity [51]; and G2+ rectal bleeding [39,82]. Additional patient predictors that were associated with late GI toxicities with varying severity were cardiovascular history [100], hemorrhoids [61], and structural geometry factors (volume of rectum/planning target volume (PTV)) [71,152]. Clinical factors such as the use of anti-coagulants or anti-aggregants were associated with G1+ GI toxicity [96] and G2+ GI toxicity [31]. Pre-treatment TURP [96] and previous abdominal or pelvic surgery [58,82,120] were also predictive. Treatment-related factors, notably dose per fraction [149], image guidance [103], pelvic field [196], RT technique [100], and use of fiducial markers all influenced late GI toxicities to different levels of severity [165]. Radiomic and principal component analysis features (e.g., damage integrated over rectal surface) [40,97] were occasionally reported as predictors for rectal bleeding.

For patients treated with MHRT (Table 3), predictors for late GI toxicities were primarily dosimetric, with patient and clinical factors showing significant associations. No treatment factor was identified. Dosimetric factors, specifically rectal dose metrics, were predominant predictors across multiple GI endpoints. Rectal dose parameters, including V40–66, D0.1cc, and Dmax, significantly predicted G1–2 GI toxicity in multivariate analysis [85,183], while rectal dose (V70) was also associated with this endpoint [110]. Similarly, rectal dose (V30–90) was strongly predictive of G2+ rectal bleeding [164]. Other endpoints such as fecal incontinence, proctitis, tenesmus, mucosal loss, bowel urgency, loose stool, bowel distress, and crampy abdominal pain were also associated with intermediate rectal dose metrics (V43–59), consistently identified in univariate analyses [164].

In the UHRT cohort, late gastrointestinal (GI) toxicities demonstrated similar predictive patterns as observed previously in the CFRT and moderate hypofractionation cohorts (Table 3). Dosimetric predictors primarily involved rectal dose metrics. Rectal doses, specifically parameters representing high-dose regions such as D0.1cc, D0.5cc, and D1cc, were significant predictors of G2+ GI toxicity in multivariate analysis [17]. Additionally, rectal dose metrics (V35–40, D1cc, D2cc, D5cc, Dmax, Dmean) significantly predicted G1+/2+ rectal toxicity [169], while rectal dose at V38 was associated with G2+ rectal toxicity [169]. Similarly, rectal dose at V38–40 was predictive of G2+ rectal bleeding [84]. Patient-specific factors were also notable predictors. Acute G2+ GI toxicity, acute bowel symptoms, and higher baseline bowel sub-domain scores significantly predicted late G2+ GI toxicity [191], as well as the presence of predicted G2+ rectal bleeding [84]. Treatment-related factors were significant predictors for late G2+ rectal bleeding, with increased treatment volumes, wider PTV margins, and higher prescription doses identified as risk factors [84]. Clinically, the use of anti-coagulants was also associated with increased risk of late G2+ rectal bleeding [84].

### 3.3. Predictors of Genitourinary Toxicities

#### 3.3.1. Acute Genitourinary Toxicities

In the CFRT setting (Table 4), multiple factors were associated with acute GU toxicities: bladder V14–27 was linked to acute G1+ toxicity [49], while higher bladder subregion doses (V56–71, Dmean and V80) were reported for G2+ toxicity, urinary frequency, and incontinence [32,129,167]. Urethra V74 and V71 also predicted urinary frequency and incontinence [32,167,171]. Among patient factors, smoking habit [129,161] and baseline urinary function [167,171] were observed. Clinical parameters such as pre-treatment/mid-course TGF-β1 [178], TURP [171], and use of anti-hypertensives [171] contributed to these outcomes. Additional associations were noted for radiomic features [129,193], structural geometry [161,182], and prostate volume [167,171], indicating a range of dosimetric and patient-specific factors in predicting acute GU toxicity.

In the MHRT setting (Table 4), multiple factors were reported as predictors of acute GU toxicities: a higher IPSS pretreatment score was associated with an overall increase in GU toxicity [63], while irradiation of seminal vesicles/pelvic lymph nodes was linked to G1–2 toxicity [110]. For G2+ GU toxicity, bladder dose (V40–50) [110] and prostate volume [193] were identified, as well as the use of anti-aggregants/anti-coagulants [29] and radiomic features [193]. Bladder dose (V52–70) was additionally implicated in acute G2+ urinary toxicity [107,146]. Notably, baseline IPSS was predictive of IPSS 15+, with smoking and bladder subregion dose (V50–70) also contributing [140], underscoring that pre-existing urinary conditions may exacerbate acute symptom severity.

In the UHRT setting (Table 4), several factors were associated with acute G2 GU toxicity, including age [175], baseline GU toxicity [175], dose escalation [175], risk group [28], and bladder Dmean (1031) [175]. For G2+ GU toxicity, significant predictors encompassed baseline IPSS/IPSS-QoL [160,179,199], bladder volume [160], age [199], bladder dose [160,179,199], and prostate volume [186,199]. An additional endpoint, IPSS total score +10, or initiation of alpha blockers was linked to bladder/bladder wall dose (V10–35, D5cc, Dmean) [184].

#### 3.3.2. Late Genitourinary Toxicities

There are considerably more publications attempting to predict late GU toxicities (Table 5). Multiple predictors of late GU toxicities following CFRT were identified, with dosimetric factors being the most reported, followed by patient and clinical factors. Notably, the bladder and urethra were the two organs with dosimetric factors most frequently linked to late GU toxicity, with both whole-organ and subregional doses demonstrating predictive value [32,128,150,156,167]. For bladder dose, significant associations were observed across multiple endpoints. Bladder surface/wall dose (V80) was a key predictor of late G1+ toxicity [98,108,156,189], while whole bladder or bladder wall subregion doses (V55–80, Dmean) were predictive of late G2+ toxicity [47,76,94,129]. Additionally, bladder/bladder neck subregion doses (V48–75, Dmean) and a urethral dose of V71 were linked to late hematuria [32,128,150,156,167], evident in both univariate and multivariate analysis. Late urinary retention was also associated with the bladder or bladder wall subregion dose (V10–82, Dmean) [32,47,167]. These findings indicate that both bladder and urethra dosimetry are closely related to late GU toxicity in CFRT patients. Among patient factors, age was a recurrent predictor under multivariate analysis for G2+ toxicity [94,191], G3+ toxicity [201], urinary retention [47], and incontinence [171]. Prostate volume was also identified as a predictor for late G1+ GU toxicity and was included in structural geometry factors influencing urinary retention. A prominent finding was that clinical factors, such as previous GU toxicity status during and after treatment, strongly predicted the late GU toxicity. This review identifies that baseline, acute urinary, acute hematologic, or rectal toxicity have been reported by multiple studies as predictors of late G2+ GU toxicity [47,94,151,161,185,191]. Acute urinary toxicity was also linked to G1+ toxicity [47,64]. Additionally, the dose escalation was associated with increased late G1+ GU toxicity, with higher prescription dose (70.2 Gy vs. 79.2 Gy) being a predictor of G2+ toxicity [185] and radiotherapy field size retaining significance in multivariate analysis [136,196]. These findings highlight the dominant role of bladder and urethra dosimetry, particularly subregional dose effects, along with age, prostate volume, and baseline/acute toxicity measures, in predicting late GU toxicities following CFRT.

In the MHRT cohort (Table 5), similar patterns were observed in the predictors of late GU toxicities, with dosimetric factors being the most associated. Bladder dose (V60–75) was significantly associated with late G2 GU toxicity [110], while bladder dose (V10) was a predictor of G2+ GU toxicity [194]. Additionally, bladder/bladder wall dose (V17–57) was linked to G2+ urinary toxicity [107]. Surface dose statistics of the bladder were also viewed as a significant predictor for patients scoring IPSS ≥ 15 [108]. For cystitis, radiomic features were identified, suggesting potential associations with textural variations in dose distributions [77]. Patient-related factors continued to play a significant role. Pre-treatment GU symptoms [110] and acute GU toxicity [146] were predictors of late G2 GU toxicity, reinforcing the trend observed in CFRT that baseline and acute symptoms strongly predict late toxicity. Clinical factors also contributed, with pre-treatment TURP associated with late G2 GU toxicity [180], and high-dose amifostine linked to urinary frequency [77]. Additionally, the use of anti-hypertensives [163] and baseline IPSS [108] were predictors of IPSS 15+, indicating that pre-existing urinary conditions influence post-treatment symptom severity.

In the UHRT cohort (Table 5), unlike the CFRT and MHRT cohorts, prostate volume was the most frequently reported disease factor linked to late G2+ GU [123,157,179,191]. Bladder and urethral dose metrics remained critical dosimetric predictors: bladder V35–40, Dmax, and D1/2/5cc were associated with G1+ GU toxicity [169] while urethra V42–44, Dmax, and maximum urethral dose metric (MUDM) predicted G2+ and G3+ GU toxicities [123,157,179]. Bladder V28–40, D0.5/1/5cc, Dmax [17,169], and prostate dose (V46–50) [157] also emerged as significant for late G2+ toxicity. Additionally, treatment-related factors were more commonly noted in UHRT than in CFRT or MHRT, with treatment machine, fiducial use, and treatment duration influencing G2+ GU toxicity [157,177,191]. Age also showed predictive value for G2+ toxicity [179] and late urinary flare [138]. Baseline or acute GU toxicity (IPSS, EPIC-26) remained crucial [17,191]. A unique observation in UHRT was genetic predisposition (mirSNPs) predicting G2+ GU toxicity [177]. Lastly, bladder dose (V85–100, D2/10cc, Dmean) correlated with quality-of-life reductions in urinary irritation [142].

### 3.4. Predictive Models

Predictive models based on ML or ANN for GI and GU toxicities were occasionally reported in the reviewed literature (Table 6). In the CFRT cohort, stacking algorithms combined with elastic-net regression provided moderate predictive performance (AUC ranging 0.65–0.77) for acute GI and GU toxicities, integrating clinical (e.g., rectal dose parameters, bladder volumes) and radiomic features (e.g., Gray Level Dependence Matrix (GLDM), Gray-Level Size Zone Matrix (GLSZM)) [170]. Notably, a RF model significantly outperformed other approaches (area under curve (AUC) = 0.95) for predicting acute G1+ cystitis, using comprehensive radiomic and clinical parameters (tumor stage, grade, run-length matrix, entropy, gray-level variance) [200]. An artificial neural network (ANN) model for late G2 rectal bleeding integrating clinical and dosimetric features demonstrated good accuracy (AUC = 0.714) [89], yet was lower than the random forest (RF) model performance for acute endpoints. Within the MHRT cohort, a feasibility study predicting acute G2–3 GI and GU toxicities adopted the ANN method as well, based on clinical and dosimetric features (mean square error: 1.22–1.62) [69]. Direct comparison with CFRT or combined models was limited due to differences in reporting metrics. In the UHRT cohort, an interactive grouped greedy algorithm (IGA) utilizing pelvic dosimetric parameters yielded the lowest reported predictive performance (AUC = 0.57) for acute G2+ GU toxicity [198]. Models derived from combined CFRT and MHRT cohorts showed mixed outcomes. ANN and support vector machine (SVM) models predicting combined acute G2–4 GI and GU toxicities reported moderate predictive capabilities (ANN AUC = 0.697; SVM AUC = 0.717) [80]. However, a RF model explicitly targeting acute G2+ GI toxicity demonstrated excellent accuracy (AUC = 0.95) [197], comparable to the high-performing CFRT cystitis model, leveraging a focused selection of rectal dose (Dmax, Dmean, V35–65, D70–76 Gy) and anatomical parameters (prostate and rectal volumes) [200]. Predictive modeling for late GI toxicities within combined cohorts revealed strong predictive capacity as well, with the ANN and least absolute shrinkage and selection operator (LASSO) models achieving robust performance (AUC = 0.71–0.77) for G1+ late fecal incontinence [158], primarily driven by rectal dosimetry and clinical factors such as abdominal surgery, antihypertensives, and anti-coagulants.

## 4. Discussion

To our knowledge, this is the first scoping review to identify all of the current literature on predictors and predictive models for all acute and late outcomes of GI and GU toxicities, in all three fractionation schemes (i.e., CFRT, MHRT, and UHRT), in PCa patients. The findings reveal a complicated and multifaceted interplay between major factors such as dosimetric, patient, clinical, treatment, and disease factors in determining toxicity risks.

### 4.1. Predictors of GI and GU Toxicities

Among all reviewed studies, dosimetric factors are the most selected predictors. For instance, rectum or rectal sub-region dose is most predictive of acute G2+ rectal and late G1+ rectal bleeding toxicity; bladder or bladder sub-region dose is most predictive of acute GU toxicities. Interestingly, patient factors such as baseline or acute urinary toxicity are the most selected for late G2+ GU toxicities. However, it should be appreciated that toxicities are multifactorial in nature. Hence, a set of predictors should be utilized to model a toxicity outcome, as systematically consolidated by this scoping review.

### 4.2. Performance of Prediction Models

Diversified types of models are reviewed, including stacking ensembles that merge clinical, dosimetric, and radiomic features via elastic net regularization; ANNs processing nonlinear relationships for classification and regression; Random Forests excelling in high-dimensional radiomic data; and SVMs optimizing feature selection in smaller cohorts, while LASSO prioritizes parsimony via linear regression. The models were assessed by the AUC for binary classification and MSE for regression. Metric heterogeneity, such as ANNs omitting the AUC, limits comparability. While the AUC evaluates discrimination, the MSE quantifies regression error. Standardizing metrics is required to strengthen clinical utility. The reviewed predictive models for GI and GU toxicities demonstrated satisfactory performance, with over half achieving an AUC above 0.70. Performance ranged significantly, from an IGA model (AUC = 0.57) to RF models (AUC up to 0.95). Dosimetric parameters, especially doses to the rectum, urethra, and bladder, were most used. CFRT models outperform MHRT and UHRT models in this review, hinting at challenges in generalization across treatment protocols and patient populations.

Although traditional DVH parameters remain widely used, incorporating 3D dose distribution has improved prediction accuracy and classification, overcoming limitations of DVH-based approaches, such as ignoring spatial dose variations and assuming uniform radiosensitivity in organs at risk [117,121].

In the study comparing the performance of dosimetric-only, dosimetric–radiomic, and radiomic models [170], adding radiomic variables to dosimetric features may improve the performance of predictive models, despite the opposite trend also being observed [170,197]. Further investigation on radiomic texture features would be required [170].

With the advancement in computational power and neural network development, more ANN and radiomic models are being developed with satisfactory performance in multiple models [69,80,89,158,170,200]. However, the heterogeneous distribution of training data of severe toxicity grades (RTOG/CTCAE G3 and G4) leads to biased training, particularly for ML and ANN, requiring high-quality training data [80].

Predictive models incorporating clinical parameters showed superior performance [80,158,197,200]. Some studies highlighted the importance of including clinical variables in models [158,197]. Specific variables such as previous surgery, which may increase tissue sensitivity due to inflammation [89,158], and the use of statin drugs alongside initial PSA levels [197], were noted as significant. The integration of multidimensional predictors—such as dosimetric, clinical, and radiomic features—is essential for developing models that comprehensively capture factors associated with toxicity outcomes. However, successful clinical translation requires rigorous multi-institutional validation, as well as standardized protocols for data collection and model development to ensure generalizability. To be clinically useful, these models should be designed for seamless integration into the radiotherapy workflow, enabling risk stratification for EBRT-related toxicities prior to or during treatment planning. For example, such models could support decision-making when selecting among fractionation schemes with equivalent disease control efficacy.

### 4.3. Limitations on Toxicity Prediction Studies

Heterogeneity in toxicity scoring has been observed. Although 40.2% of the reviewed literature adopted RTOG and the same amount used the CTCAE scale, a fundamental difference in the grading exists. RTOG scales adopt a combined grading approach for several symptoms and provide an overall grading. In contrast, individual symptom grading is utilized in CTCAE scales. Notably, both scales could yield significantly different results. In the PACE-C trial’s preliminary results comparing MHRT and UHRT toxicities, the RTOG scale revealed no significant acute GU nor GI differences, except in CTCAE measurement [30]. This highlighted the potential risk of reduced sensitivity of RTOG scales in identifying significant toxicities.

Regarding predictors, dosimetric factors are the most selected. However, most of the included studies investigate the planned dose instead of the delivered dose, which usually does not account for inter-fraction motion or systematic or random setup errors. With more recent preliminary results from Shelley et al. and Ong et al. [173,181], which utilized the delivered dose, also termed as the accumulated dose to rectum, the GI toxicity predictive performances are improved. Another example of mitigating such limitations is potentially the use of simulated motion-inclusive DVH with random isotropic or anisotropic shifts [105,106]. Furthermore, most of the studies investigated only the entire rectal or bladder volume, without measuring the dosimetric factor from sub-regions.

Other major limitations include the lack of reporting details. For instance, there are occasional missing details of the detailed fractionation scheme, including fraction size, prostate risk status, image guidance modality, treatment protocol, and region of interest (ROI) definitions, including those of sub-volumes. This renders future replication or reference to previous studies difficult. The retrospective nature of most studies also inherits the limitation that most confounding factors cannot be effectively controlled in the study.

### 4.4. Recommendation

Based on the identified limitations, some recommendations are provided for future studies aiming to identify predictors for GI or GU toxicities in PCa patients receiving EBRT.

First, it is advised that the CTCAE scale should be used, as continuous updates are available with active reviews [19]. CTCAE is potentially more sensitive than RTOG scales towards toxicity detection [30] by allowing individual grading of signs and symptoms.

Second, the planned dose and accumulated or delivered dose should both be investigated in future studies. Since toxicity arises from actual treatment delivery instead of the planned treatment, any treatment-associated factors, such as inter-fraction motion or systematic or random set-up, should be considered through simulation if analyzed retrospectively. Future studies could refer to previous work that adopted similar methods [155,160].

Third, the whole volume and sub-regions or sub-volumes of the rectum, bladder, and urethra should be investigated thoroughly, as in some reviewed studies [32,85,87,173,184]. This is because the anterior portion of the rectum often receives more dose than the posterior counterpart, similarly in the bladder, but in different directions, due to proximity to the prostate gland. It is not uncommon that studies with sub-volume analysis provide reasonable predictors. For example, the trigone dose of the bladder predicts acute G2+ GU toxicity [198], the trigone dose–surface volume predicts an acute increase in IPSS score by 10 [160], the bladder wall subregion predicts acute incontinence [167], etc. Rectal or rectal wall subregions may inform late G1+ rectal bleeding [132,173]. Hence, a more comprehensive analysis should be conducted to specify which sub-region carries the highest predictive value.

Apart from investigative endpoints, it is suggested that future studies uphold consistency when reporting patients’ characteristics. A list of reporting items is proposed in Table 7. Three categories are proposed: clinical characteristics, treatment, and medication. Since this review has identified studies reporting underlying diseases such as diabetes or hypertension and baseline toxicity could be factors related to toxicity development, investigators are advised to collect and report such information. Due to rapid advancement in EBRT technology, such as online or offline image verification, immobilization devices, treatment machines such as conventional linear accelerator (LINAC), tomotherapy, and cyberknife, a basket of varieties and potential confounders in toxicity study exists. Such information should be provided to ensure comparability across studies. Similarly, it is underlined that the contouring definition of all relevant ROIs, including the prostate gland, rectum, bladder, urethra, and their sub-regions, should be provided in full. Considering that other medications such as anti-coagulants may aggravate GI toxicities and rectal bleeding in multiple reports [89,120,143,152,161] but some do not [32,61], reporting of such usage is vital to facilitate prediction analysis and control of confounders.

Implementation of prospective external validation with multicenter validation cohorts is highly encouraged for improved confounder control, a heightened level of evidence, increased robustness, and reduced selection bias. The current review, therefore, serves as a basis for inspiring the design of such research, hinting at potential predictors.

A major research gap lies in radiomic analysis, a high-throughput image feature extraction and analysis methodology, proposed by Lambin et al. [203]. Currently, only a few publications reviewed by this study investigated radiomics for GI and GU toxicity prediction in PCa. Since radiomics can be applied to various imaging modalities, including CT, MRI, and RT dose fluence maps, a vast amount of subtle imaging features not visible to human readers can be extracted and analyzed for any potential connections with toxicity. Its usage has been widely applied in prognosis prediction in PCa, such as those by Ching et al. [204] and Leung et al. [205], and head-and-neck toxicity prediction by Nicol et al. [206]. Correlations between radiomic features and toxicities warrant further investigation to facilitate personalized PCa EBRT.

### 4.5. Limitation of This Review

A limitation of this review, due to its scoping nature, is that each predictor category is not evaluated in an in-depth manner, which may produce further insights into their distribution and bias. Use of the predictive factors identified from this review is encouraged after robust prospective modelling and testing. Another limitation is that this review has not separately investigated predictors reported by studies using RTOG or CTCAE toxicity scales. Such limitations may be addressed more practically by a systematic review and meta-analysis approach after further stratification of studies.

## 5. Conclusions

This scoping review included 169 studies on acute and late GI and GU toxicities among CFRT, MHRT, and UHRT PCa cohorts. Detailed and categorized predictors have been systematically reviewed. Dosimetric parameters are most often reported as predictive factors, followed by patient and clinical factors. It is particularly recommended that future studies should be prospective in nature with external validation for confounder control, adopt CTCAE for toxicity assessment, investigate both the planned and delivered dose, define the whole volume and sub-volume of ROI, and report consistently. It is hoped that with more high-quality evidence, the development of a personalized PCa EBRT treatment strategy can be formulated.

## Figures and Tables

**Table 1:** Summary statistics of included studies.

Summary Statistic	GI	GU	Overall
Full-text articles (*N*,%)	127 (75.1)	78 (46.2)	169
Patient cohort size (median, range)	156 (9–1499)	158 (11–3243)	168 (9–3243)
RTOG reporting scale (*N*,%)	58 (45.7)	26 (33.3)	68 (40.2)
CTCAE reporting scale (*N*,%)	53 (41.7)	37 (47.4)	68 (40.2)
CFRT (*N*,%)	85 (66.9)	31 (39.7)	123 (72.8)
MHRT (*N*,%)	13 (10.2)	12 (15.4)	34 (20.1)
UHRT (*N*,%)	7 (7.1)	15 (19.2)	21 (12.4)
Univariate analysis available (*N*,%)	104 (81.9)	61 (78.2)	139 (82.2)
Multivariate analysis available (*N*,%)	68 (53.5)	41 (52.6)	94 (55.6)
Internal validation of model (*N*,%)	29 (37.2)	18 (23.1)	40 (23.7)
External validation of model (*N*,%)	5 (6.4)	1 (1.3)	5 (3.0)

GI: gastrointestinal, GU: genitourinary, RTOG: Radiation Therapy Oncology Group, CTCAE: Common Terminology Criteria for Adverse Events, CFRT: conventional fractionated radiotherapy, MHRT: moderate hypofractionated radiotherapy, UHRT: ultra-hypofractionated radiotherapy.

**Table 2:** Predictors of acute GI toxicities.

Toxicity Outcome	Predictor Category	Predictor	Univariate Analysis (*N*)	Multivariate Analysis (*N*)
G1+	Dosimetric	Rectal dose (V10–73; MHRT: Dmax; UHRT: V28)	4	1
		Principle component analysis features	1	1
	Patient	Age		1
		Rectal volume	1	1
		Hemorrhoids	1	1
		GI co-morbidities	1	1
		Alcohol consumption	1	
		Microbial alpha diversity/elevated MCPI	1	
	Clinical	TURP		1
		Previous abdominal/pelvic surgery	1	
		Hormone therapy		1
G2	Dosimetric	Rectal dose (V37–70, Dmean)		2
	Genetic	Polymorphisms (XRCC3 rs1799794 SNP)	1	1
G2+	Patient	History of diabetes mellitus	1	
	Clinical	Use of anti-coagulants	1	
		Statin medication (MHRT only)	1	
		ADT (MHRT only)	1	
	Dosimetric	Rectal dose (V70, D2cc; MHRT: V50–65; UHRT: V10–30, D25.3/50/10cc, Dmean)	4	2
		Dose region (V65)	1	
G1+ rectal toxicity	Clinical	History of diabetes mellitus	1	
		ADT	1	1
	Treatment	Pelvic nodes irradiation	1	1
	Dosimetric	Rectal dose (V60–70, Dmean; MHRT: D50 and V70)	3	1
G2 rectal toxicity	Dosimetric	Rectal dose (V60–70; MHRT: V53)	1	1
	Treatment	Hormone therapy	1	
G2+ rectal toxicity	Dosimetric	Rectum/rectal subregion dose (V70, Dmean; MHRT: V67–68)	5	2
		Structural geometry (rectum cross-sectional area/surface area/extension, PTV volume/height)	1	
	Clinical	Use of anti-coagulants	1	1
		ADT		1
	Treatment	Pelvic nodes irradiation	1	
	Patient	Hemorrhoids		1
Rectal bleeding	Patient	Hemorrhoids	1	3
	Dosimetric	Rectal dose (Dmean)		2
		Rectal dose (MHRT: V51–65) (MHRT only)	1	
Diarrhea	Dosimetric	Rectal dose (V60–75)	1	
	Patient	History of diabetes mellitus	1	1
Proctitis	Patient	Biomarkers (pro-hepcidin/IL-6/TNF/hemoglobin/ferritin/transferrin)	1	
	Clinical	High dose amifostine (MHRT only)		1
Incontinence	Patient	Age		1
Rectal urgency	Dosimetric	Rectal dose (V70)		1
	Treatment	NHT		1
	Patient	Hemorrhoids		1
Tenesmus	Treatment	Irradiation of seminal vesicle	1	1
	Dosimetric	Rectal dose (Dmean)		1
Complication requiring drugs	Treatment	Irradiation of seminal vesicle	1	
	Disease	Target volume	1	1
	Dosimetric	Rectal dose (Dmean)	1	1
Stool frequency	Treatment	ADT	1	1
		Irradiation of seminal vesicle	1	
Painful bowel movement	Patient	Hemorrhoids		1
Bowel habits	Dosimetric	Rectal dose (V70)		1
	Patient	Hemorrhoids		1

Unless specified otherwise in brackets, all predictors refer to CFRT. CFRT: conventional fractionated radiotherapy, MHRT: moderate hypofractionated radiotherapy, UHRT: ultra-hypofractionated radiotherapy, GI: gastrointestinal, MCPI: microbial community polarization index, TURP: transurethral resection of the prostate, SNP: single-nucleotide polymorphism, ADT: androgen deprivation therapy, PTV: planning target volume, NHT: neoadjuvant hormone therapy.

**Table 3:** Predictors of late GI toxicities.

Toxicity Outcome	Predictor Category	Predictor	Univariate Analysis (*N*)	Multivariate Analysis (*N*)
G1+	Dosimetric	Rectal dose (V35–70; MHRT: V70)	3	3
		Prostate subregion dose (Dmean)	1	1
	Clinical	Use of anti-hypertensives/anti-coagulants	2	1
	Patient	Acute GI toxicity	2	1
		Rectal volume		1
		Age (MHRT only)	1	
		Pretreatment GI symptoms (MHRT only)	1	
G2+	Dosimetric	Rectum/rectal subregion dose (V45–70, Dmean/0.03/50%; MHRT: V40–66, D0.1/1cc, Dmax; UHRT: D0.1/0.5/1cc)	9	5
		Principal component analysis features (MHRT only)	1	
	Patient	Age	2	3
		Age-comorbidity score		1
		Caucasian race		1
		History of myocardial infarction/congestive heart failure	1	1
		Acute/baseline GI toxicity	2	2
		Hemorrhoids		1
		Rectum volume	1	
		Prostate/prostate subregion dose (D98, isotropic expansion)	1	1
		G2+ acute GI toxicity (MHRT and UHRT only)	3	1
		Acute bowel symptoms (UHRT only)	1	
		Baseline EPIC-26 bowel sub-domain score (UHRT only)	1	
	Clinical	Use of anti-coagulants/anti-aggregants	1	1
		ADT	1	1
	Treatment	RT field (prostate + pelvic field vs. prostate only)	1	
		RT technique (3DCRT vs. IMRT)	2	1
		Evening RT timing	1	1
	Disease	Clinical staging	1	
G3+	Patient	Acute G2+ GI toxicity		2
		Age	1	1
		History of myocardial infarction/congestive heart failure	1	1
		Increasing CCMI	1	
		Age-comorbidity score		1
	Treatment	RT technique (IG-3DCRT/IG-IMRT vs. 3DCRT)	1	
G1 rectal bleeding	Dosimetric	Rectal wall dose (V6)	1	
G1+ rectal bleeding	Dosimetric	Rectum/rectal subregion dose (V40–75, Dmean, length-based integral dose; MHRT: V51–55)	8	5
		Principal component analysis features	1	1
		Damage integrated over rectal surface (cm)	1	
	Patient	Hemorrhoids		1
		Structural geometry (volume of rectum/PTV)		2
		History of cardiovascular disease	1	1
		Smoking		1
	Clinical	Previous abdominal surgery	1	1
G2 rectal bleeding	Dosimetric	Rectal dose (V90, EUD, AUC-DVH 50/80/90)	2	2
	Patient	Hemorrhoids	1	1
		Rectum size		1
	Clinical	Use of anti-coagulants/ADT	1	1
		Previous abdominal surgery	1	1
G2+ rectal bleeding	Dosimetric	Rectum/rectal subregion dose (V30–75, Dmean, Dmax, EUD; MHRT: V30–90; UHRT: V38–40)	13	5
		ICA parameter	1	
	Clinical	Previous abdominal/pelvic surgery	2	2
		Use of anti-coagulants/anti-aggregants (CFRT and UHRT)	4	2
	Patient	Structural geometry (volume of rectum/rectal wall, length of rectum/PTV, rectal area)	2	
		Age	2	2
		Acute rectal toxicity	2	1
		History of diabetes mellitus (CFRT and MHRT)	3	3
		Platelet count	1	1
		Hemorrhoids (CFRT and UHRT)	1	
	Disease	Risk group	1	
		Clinical staging (CFRT and MHRT)	3	2
		Initial PSA	1	
		Treatment volume (UHRT only)	1	
	Genetic	MicroRNAs (Ku80, miR-99a, miR-147, miR-508, miR-199b)	1	1
	Treatment	Prescription dose (CFRT and UHRT)	2	
		PTV margins (UHRT only)	1	
		RT beam geometry	1	
		Fiducial marker	1	
G3+ rectal bleeding	Clinical	Previous abdominal/pelvic surgery	1	2
G1+ rectal toxicity	Dosimetric	Rectum/rectal ring/anal wall dose (V40–70; UHRT: V35–40, D1/2/5cc, Dmax, Dmean)	3	2
	Genetic	Micronuclei indices	1	
G1–2 rectal toxicity	Dosimetric	Rectal dose (V40–60)	1	
G2 rectal toxicity	Dosimetric	Rectal dose (V70–75, Dmax)	3	1
	Treatment	Prescribed dose	1	
G2+ rectal toxicity	Dosimetric	Rectum/rectal subregion dose (V50–75, Dmean, Dmedian, EUD; UHRT: V35–40, D1/2/5cc, Dmax, Dmean)	7	4
	Clinical	Use of anti-coagulants/anti-aggregants		1
	Patient	Acute rectal toxicity/diarrhea/tenesmus/any rectal symptoms	1	1
		Rectum volume	1	
		Caucasian race		1
		History of cardiovascular disease	1	
	Genetic	Polymorphism (VEGF -7C > T, ATTGT haplotype)	1	1
	Disease	Tumor risk group	1	
	Treatment	Prescribed dose/dose per fraction	1	1
		RT technique (3DCRT vs. IMRT)	1	1
Fecal incontinence	Dosimetric	Rectal dose (V15–75; MHRT: Dmean)	5	3
		Dose of anal sphincters, iliococcygeal muscle, levator ani muscle (V15–55)	1	1
	Patient	Acute G2+ fecal incontinence	1	3
		Previous bowel symptoms	1	
		History of diabetes mellitus	1	1
		Previous diseases of the colon (CHRT and MHRT)	2	
		Hemorrhoids		1
	Clinical	Previous abdominal/pelvic surgery (CFRT and MHRT)	3	3
		Use of anti-hypertensive		1
Stool frequency	Dosimetric	Rectal dose (V60–65, EUD)	1	1
		Dose of iliococcygeal muscle/puborectalis muscle/levator ani muscle (V40–45, Dmean, EUD)	1	1
	Patient	Age	1	1
		Acute complaint	1	1
		Presence of cardiovascular diseases	1	1
		Baseline stool frequency	1	1
		G2+ acute GI toxicity	1	
	Treatment	ADT before RT		1
	Clinical	Previous abdominal/pelvic surgery		1
Tenesmus	Dosimetric	Rectum/rectal subregion dose (V50–65; MHRT: V51–59)	1	1
	Patient	Rectum volume	1	
	Clinical	Previous abdominal/pelvic surgery		1
Abdominal pain	Dosimetric	Rectal dose (V50–70; MHRT: V43)	1	1
	Patient	Chronic renal failure		1
	Treatment	RT technique (3DCRT vs. IMRT)	1	
	Clinical	Previous abdominal/pelvic surgery		1
Proctitis	Dosimetric	Rectum/rectal subregion dose (V50–70, EUD; MHRT: V59)	4	1
	Patient	Acute rectal toxicities/endoscopic proctitis/clinical proctitis	2	2
		Age	1	1
	Treatment	RT planning constraints 3rd criteria	1	
		RT technique		1
Diarrhea	Dosimetric	Rectal dose (V50)	1	
	Treatment	RT technique (IGRT vs. Non-IGRT)	1	1
Bowel/rectal urgency	Dosimetric	Rectal dose/rectal subregion dose (V50–75; MHRT: V59)	1	1
	Patient	Chronic renal failure		1
		Acute complaint	1	
		Hemorrhoids		1
	Treatment	RT technique (IGRT vs. Non-IGRT)	1	1
Mucosal loss	Dosimetric	Rectal dose (V60–65; MHRT: V51–59)	1	
	Patient	Acute complaint	1	
Underwear soil	Dosimetric	Rectal subregion dose (V75)		1
	Patient	Acute complaint	1	1
		Smoking		1
Rectal pain	Treatment	RT technique (IGRT vs. Non-IGRT)	1	1
	Patient	G2+ acute GI toxicity	1	1
	Dosimetric	Rectal dose (EUD)		1
Loose stools	Dosimetric	Rectum/rectal subregion dose (DSH V23; MHRT: V43–59)	2	1
Involuntary gas discharge/strain upon defecation	Dosimetric	Rectal subregion dose (V50–75)		1
Bowel distress	Dosimetric	Rectal dose (V59) (MHRT only)	1	
Change in bowel habits	Treatment	RT technique (IGRT vs. Non-IGRT)	1	1
		Spontaneous gaps and breaks		1
	Patient	Chronic renal failure		1
		Hemorrhoids		1
		% of early apoptotic cells	1	1
		Higher spontaneous chromatid aberration yield	1	
	Dosimetric	Rectal dose (V50)	1	

Unless specified otherwise in brackets, all predictors refer to CFRT. CFRT: conventional fractionated radiotherapy, MHRT: moderate hypofractionated radiotherapy, GI: gastrointestinal, EPIC-26: expanded prostate cancer index composite, ADT: androgen deprivation therapy, RT: radiotherapy, 3DCRT: three-dimensional conformal radiotherapy, IMRT: intensity-modulated radiotherapy, CCMI: Charlson comorbidity index, IG-3DCRT: image-guided 3D conformal radiotherapy, IG-IMRT: image-guided intensity-modulated radiotherapy, PTV: planning target volume, EUD: equivalent uniform dose, AUC-DVH: area under curve–dose volume histogram, UHRT: ultra-hypofractionated radiotherapy, ICA: independent component analysis, PSA: prostate-specific antigen, RNA: ribonucleic acid, VEGF: vascular endothelial growth factor, IGRT: image-guided radiotherapy, DSH: dose surface histogram.

**Table 4:** Predictors of acute GU toxicities.

Toxicity Outcome	Predictor Category	Predictor	Univariate Analysis (*N*)	Multivariate Analysis (*N*)
Increase in GU toxicity	Patient	IPSS pretreatment score (MHRT only)		1
G1+	Dosimetric	Bladder dose (V14–27; MHRT: V40–50)	1	2
	Clinical	Pre-treatment/mid-course TGF-β1 concentration	1	
G1–2	Treatment	Irradiation of seminal vesicle/pelvic LNs (MHRT only)	1	
G2	Patient	Age (UHRT only)	1	
		Baseline GU toxicity (UHRT only)	1	1
	Treatment	Dose escalation (UHRT only)	1	
	Disease	Risk group (UHRT only)	1	
	Dosimetric	Bladder Dmean (UHRT only)	1	1
G2+	Patient	Smoking habit	2	1
		Structural geometry (volume of bladder/PTV)	2	
		Baseline IPSS/IPSS-QoL (UHRT only)	3	1
		Bladder volume (UHRT only)	1	
		Age (UHRT only)	1	1
	Dosimetric	Bladder dose (V80; UHRT: EQD2 = 10, MUDM)	2	1
	Radiomic features	CBCT features (bladder): NGTDM coarseness/strength, GLSZM LZHGE	1	
		GLRLM-GLN, GLSZM-ZSN, GLSZM-ZSV, global kurtosis (MHRT only)	1	
	Clinical	Use of anti-aggregants/anti-coagulants (MHRT only)	1	
	Disease	Prostate volume (MHRT and UHRT)	3	1
G2+ urinary toxicity	Dosimetric	Bladder dose (V52–70) (MHRT only)	1	
Dysuria	Patient	Age	2	
	Clinical	Use of anti-hypertensives	1	
	Disease	Prostate volume	1	
Urinary frequency	Clinical	TURP	1	
		Baseline retention/frequency	1	
Urinary retention	Dosimetric	Bladder/bladder subregion dose (V56–71, Dmean)	2	
		Urethral dose (V74)	1	
	Clinical	TURP	1	
	Patient	Baseline retention	1	
Hematuria	Clinical	TURP	2	
		Previous abdominal surgery	1	
		Use of anti-coagulants	1	
Incontinence	Dosimetric	Bladder/bladder subregion dose (V71, Dmean)	1	
		Urethral dose (V71)	1	
IPSS total score + 10 OR start alpha blockers	Dosimetric	Bladder/bladder wall dose (V10–35, D5cc, Dmean) (UHRT only)	1	1
IPSS 15+	Patient	Baseline IPSS (MHRT only)		1
		Smoking (MHRT only)		1
	Dosimetric	Bladder subregion dose (V50–70) (MHRT only)	1	1

Unless specified otherwise in brackets, all predictors refer to CFRT. CFRT: conventional fractionated radiotherapy, GU: genitourinary toxicity, IPSS: international prostate symptom score, MHRT: moderate hypofractionated radiotherapy, TGF-β1: transforming growth factor beta, LN: lymph nodes, UHRT: ultra-hypofractionated radiotherapy, PTV: planning target volume, IPSS-QoL: International Prostate Symptom Score QoL index, EQD2: equivalent Dose in 2-Gy fractions, MUDM: maximum urethral dose metric, CBCT: cone beam computed tomography, NGTDM: neighboring gray tone difference matrix, GLSZM: gray level size zone, LZHGE: large zone high gray-level emphasis, GLRLM-GLN: gray-level run-length matrix–gray-level non-uniformity, GLSZM-ZSN: gray-level size zone matrix–zone size non-uniformity, GLSZM-ZSV: gray-level size zone matrix–zone size variance, TURP: transurethral resection of the prostate.

**Table 5:** Predictors of late GU toxicities.

Toxicity Outcome	Predictor Category	Predictor	Univariate Analysis (*N*)	Multivariate Analysis (*N*)
G1+	Dosimetric	Bladder surface/bladder wall/bladder subregion dose (V80; UHRT: V35–40, Dmax, D1/2/5cc)	4	3
	Patient	Acute urinary toxicity	2	
		Baseline IPSS	1	2
	Clinical	Use of anti-hypertensives		1
	Disease	Prostate/PTV volume	1	
	Treatment	RT technique	1	
G2	Dosimetric	Bladder dose (V60–75) (MHRT only)	1	1
	Clinical	Pre-treatment TURP (MHRT only)	1	
	Patient	Pretreatment GU symptoms (MHRT only)	1	
		Acute GU toxicity (MHRT only)	1	
G2+	Patient	Baseline/acute urinary/hematologic/rectal toxicity (EPIC-26, IPSS) (CFRT and UHRT)	6	4
		Age (CFRT and MHRT)	2	2
		History of diabetes/smoking	3	1
	Dosimetric	Bladder/bladder wall dose (V55–80, Dmedian, EUD; MHRT: V10; UHRT: V28–40, D0.5/1/5cc, Dmax)	6	3
		Urethral dose (V42–44, Dmax, MUDM) (UHRT only)	3	1
		Dose region volume (V73)	1	
		Prostate dose (V46–50) (UHRT only)	1	1
		Homogeneity index V120% (UHRT only)	1	
		Prescription isodose line (UHRT only)	1	
	Clinical	TURP	3	1
		ADT	1	
	Disease	Clinical staging	1	
		Prostate/PTV volume (CFRT and UHRT)	5	4
	Treatment	RT field size	2	1
		Prescription dose (70.2 Gy vs. 79.2 Gy)	1	
		RT technique (IMRT vs. 3DCRT)	1	
		SBRT modality (UHRT only)	1	
		Fiducial use (UHRT only)		1
		Treatment duration (UHRT only)		1
	Genetic	mirSNPs (CFRT and MHRT)	1	
G3+	Patient	Age	1	
		Acute urinary/hematologic toxicity	1	1
	Disease	Prostate/PTV volume (CFRT and UHRT)	2	1
	Dosimetric	Bladder/bladder wall dose (V10–82)	1	1
		Urethral dose (MUDM) (UHRT only)	1	1
G2+ urinary toxicity	Dosimetric	Bladder/bladder wall dose (V17–57) (MHRT only)	1	
Dysuria	Dosimetric	Bladder/bladder subregion dose (Dmean, V64–68)	4	
		Urethral dose (V70)	1	
Urinary retention	Dosimetric	Bladder/bladder wall/bladder subregion dose (V10–82, Demean)	3	
		Urethral dose (V67)	1	
	Patient	Structural geometry (volume of bladder/bladder wall/prostate/PTV, bladder length)	2	
		Baseline retention	1	
		Age	1	
		Acute urinary/hematologic/rectal toxicity	1	
	Clinical	Previous abdominal surgery	1	
		Use of anti-hypertensives	1	
Hematuria	Dosimetric	Bladder/bladder wall/bladder neck/bladder subregion dose (V48–75, Dmean)	5	1
		Urethral dose (V71)	1	
	Disease	Clinical staging	1	
Incontinence	Patient	Age	1	
		TURP	1	
		History of diabetes mellitus	1	
	Clinical	Use of anti-coagulants	1	
	Dosimetric	Bladder subregion dose	1	
Urinary frequency	Patient	Age	1	
		History of diabetes	1	
		Baseline frequency	1	
		Use of anti-hypertensives/ADT	1	
		Bladder dose (R39)	1	
		High dose amifostine (MHRT only)	1	
Cytitis	Radiomic features	S5.0SumVarnc, S2.2SumVarnc, S1.0AngScMom, S0.4SumAverg, S5._5InvDfMom, WavEnHL_sN3, S4._4Contrast, S0.4InvDfMom, S4._4DifVarnc, S5._5AngScMom, S5._5DifEntrp, S3._3DifEntrp, S4._4SumOfSqs, S3.3SumVarnc, Perc.01, S4.4SumAverg, S3.3Correlat, S3.3SumAverg (MHRT only)	1	
Late urinary flare	Patient	Age (UHRT only)	1	1
QOL reduction in urinary irritation	Dosimetric	Bladder dose (V85–100, D2/10cc, Dmean) (UHRT only)	1	
Erectile dysfunction	Treatment	Hormonal therapy scheme (NHT+HT vs. NHT only)		1
		RT technique (IMRT vs. 3DCRT)	1	
IPSS ≥ 15	Clinical	Use of anti-hypertensives (MHRT only)		1
	Patient	Baseline IPSS (MHRT only)		1
	Dosimetric	Bladder dose (surface V80) (MHRT only)		1

Unless specified otherwise in brackets, all predictors refer to CFRT. CFRT: conventional fractionated radiotherapy, GU: genitourinary toxicity, UHRT: ultra-hypofractionated radiotherapy, IPSS: international prostate symptom score, PTV: planning target volume, RT: radiotherapy, MHRT: moderate hypofractionated radiotherapy, TURP: transurethral resection of the prostate, EPIC-26: expanded prostate cancer index composite-26, EUD: equivalent uniform dose, ADT: androgen deprivation therapy, IMRT: intensity-modulated radiotherapy, 3DCRT: three-dimensional conformal radiotherapy, SBRT: stereotactic body radiation therapy, mirSNPs: microRNA-related single nucleotide polymorphisms, MUDM: maximum urethral dose metric, NHT: neoadjuvant hormone therapy, HT: hormone therapy.

**Table 6:** Prediction models.

Fractionation	Toxicity Timeframe	Toxicity Outcome	Model Type	Model Features	Testing AUC
CFRT	Acute	G1+ GI toxicity	Stacking algorithm and elastic net (clinical model)	Rectal wall: Min/max/modal dose, V60	0.66
			Stacking algorithm and elastic net (clinical-radiomics model)	CT features (rectal wall): Shape-Elongation, first order, GLRLM, modal dose	0.65
			Stacking algorithm and elastic net (radiomics only model)	CT features (rectal wall): GLDM, GLSZM	0.71
	Late	G2 rectal bleeding	ANN	EUD, abdominal surgery, hemorrhoids, anti-coagulants, ADT	0.714
	Acute	G1+ GU toxicity	Stacking algorithm and elastic net (clinical model)	PTV D95, bladder volume, mean/median dose, D60/55	0.67
			Stacking algorithm and elastic net (clinical-radiomics model)	CT features (bladder wall): Shape, first order, GLCM, median dose, D40, V45	0.77
			Stacking algorithm and elastic net (radiomics only model)	CT features (bladder wall): GLDM, GLRLM, GLSZM	0.71
	Acute	G1+ cystitis	RF	Stage, grade, MRI features (bladder wall): RLN, strength, LAE, 10 percentiles, IDMN, run percentage, run entropy, GLN, correlation, gray level variance	0.95
MHRT	Acute	G2–3 GI toxicity	ANN	Age, risk group, monotherapy or not, prescription volume, RT days, rectum D30%/D60%, volume of rectum/PTV	AUC N/A (MSE = 1.62)
	Acute	G2–3 GU toxicity	ANN	Age, risk group, monotherapy or not, prescription volume, RT days, rectum D30%/D60%, bladder D50%, volume of rectum/PTV/bladder	AUC N/A (MSE = 1.22)
UHRT	Acute	G2+ GU toxicity	IGA	CTV/urethra/bladder wall/rectal wall/rectum/trigone dose V1.2–44.1	0.57
CFRT and MHRT	Acute	G2–4 GI and GU toxicity	ANN	Age, risk group, TURP, HT, prescription, field, RT days, IGRT, bladder D50%, volume of bladder/rectum/PTV	0.697
			SVM	Age, risk group, TURP, HT, prescription, field, RT days, IGRT, rectum D30%/D60%, bladder D50%, volume of bladder/rectum/PTV	0.717
CFRT and MHRT	Acute	G2+ GI toxicity	RF	Rectum Dmax/Dmean/V35–65/D70–76 Gy, prostate weight, rectal volume	0.95
	Late	G1+ late fecal incontinence	ANN	Rectum Dmean, abdominal surgery, anti-coagulants, anti-hypertensives, HT	0.77
			LASSO	Antihypertensives, abdominal surgery, colon diseases	0.71

CFRT: conventional fractionated radiotherapy, GI: gastrointestinal, CT: computed tomography, GLRLM: gray-level run-length matrix, GLDM: gray-level dependence matrix, GLSZM: gray-level size zone matrix, ANN: artificial neural network, EUD: equivalent uniform dose, ADT: androgen deprivation therapy, GU: genitourinary, PTV: planning target volume, GLCM: gray-level co-occurrence matrix, RF: random forest, MRI: magnetic resonance imaging, RLN: run length non-uniformity, LAE: large area emphasis, IDMN: inverse difference moment normalized, GLN: gray-level non-uniformity, MHRT: moderate hypofractionated radiotherapy, UHRT: ultra-hypofractionated radiotherapy, IGA: interactive grouped greedy algorithm, CTV: clinical target volume, TURP: transurethral resection of the prostate, HT: hormone therapy, IGRT: image-guided radiotherapy, SVM: support vector machine, LASSO: least absolute shrinkage selection operator, HT: hormonal therapy.

**Table 7:** Prostate cancer patient characteristics reporting items (PCPCRI).

Category	Item
Clinical Characteristics	Age (years) Weight (kg) BMI PSA (ng/dL) AJCC clinical TNM stage Diabetes (yes/no) Hypertension (yes/no) Hypercholesterolemia (yes/no) Underlying cardiovascular adverse event/disease (yes/no) Smoking (pack-year) Drinking (unit) Baseline GU toxicity (CTCAE v5 or above) Baseline GI toxicity (CTCAE v5 or above) Prostate volume (cm^3^)
Treatment	History of abdominal/pelvic surgery (yes/no) History of transurethral resection of prostate (TURP) (yes/no) RT photon energy RT fractional dose (Gy) RT total dose (Gy) RT duration (days) and schedule (daily, every other day) RT techniques (IMRT, IGRT, LINAC, TOMO, CK, MR-LINAC, US-guidance) RT treatment setup (supine/prone, immobilization device) RT prescription point (V*x*D*x*) RT treatment positioning tolerance (directions, mm) Use of hydrogel (yes/no) Use of MRI for target delineation (yes/no) Use of MRI for OAR delineation (yes/no) Use of MRI for treatment position verification (yes/no) Adaptive treatment (online, offline, no) CTV extent (whole prostate, proximal SV, whole SV, PLNs) OAR contouring definition (superior, interior, anterior, posterior, and lateral borders) RT dose calculation algorithm
Medication	ADT (drug type) ADT scheme (neoadjuvant and/or concurrent and/or adjuvant) ADT duration (months) Anti-coagulant (yes/no) Antiaggregant (yes/no) Any other medication for underlying diseases

BMI: body-mass index, PSA: prostate-specific antigen, AJCC: American Joint Committee on Cancer, TNM: tumor, node, metastasis, GU: genitourinary, GI: gastrointestinal, CTCAE: Common Terminology Criteria for Adverse Events, RT: radiotherapy, Gy: Gray, IMRT: intensity-modulated radiation therapy, IGRT: image-guided radiation therapy, LINAC: linear accelerator, TOMO: tomotherapy, CK: Cyberknife, MR-LINAC, magnetic resonance LINAC, US: ultrasound, VxDx: volume receiving x dose, OAR: organs at risk, CTV: clinical target volume, SV: seminal vesicles, PLNs: pelvic lymph nodes, ADT: androgen deprivation therapy.

## Data Availability

The data presented in this study are available in the Supplementary Materials.

## Supplementary Materials

The following supporting information can be downloaded at: https://www.mdpi.com/article/10.3390/diagnostics15111331/s1, Table S1: Toxicity Overview; Table S2: Acute GI Incidence; Table S3: Late GI Incidence; Table S4: Acute GU Incidence; Table S5: Late GU Incidence; Table S6: Acute GI CFRT; Table S7: Acute GI MHRT; Table S8: Acute GI UHRT; Table S9: Late GI CFRT; Table S10: Late GI MHRT; Table S11: Late GI UHRT; Table S12: Acute GU CFRT; Table S13: Acute GU MHRT; Table S14: Acute GU UHRT; Table S15: Late GU CFRT; Table S16: Late GU MHRT; Table S17: Late GU UHRT; Table S18: PRISMA ScR checklist.

## Appendix A

**Table A1:** Search strategy.

Database	Search String	N Results
Embase	(((Urinary AND (frequen* OR urgen* OR retent* OR pain OR bleed* OR difficul* OR irritat* OR incontinence)) OR (Urethra* AND (structure OR obstruct*)) OR Dysuria OR Nocturia OR haematuria OR hematuria OR (Diarrhoea OR diarrhea OR Tenesmus OR (Rectal AND (pain OR bleed*)) OR Proctitis OR Incontinence OR Intestinal Toxicity) OR (“toxicit*” OR morbidity OR “side effect*” OR “complication*” OR “adverse effect*” OR “adverse event*” OR “symptom*”)):ti,kw) AND (((“radiotherap*” OR “radiation therap*” OR “stereotactic body*” OR “volumetric modulated arc therapy” OR “intensity modulated” OR “conformal radiotherapy” OR “3DCRT” OR “CRT” OR “SABR”) AND (radiomic* OR feature* OR predict* OR model* OR correlat* OR corresp* OR depend* OR assoc* OR relat* OR interact* OR link* OR “risk factor*” OR analy*) AND (prostate AND (cancer OR adenocarcinoma OR carcinoma))):ti,kw)	706
Web of Science	TI = ((Urinary AND (frequen* OR urgen* OR retent* OR pain OR bleed* OR difficul* OR irritat* OR incontinence)) OR (Urethra* AND (structure OR obstruct*)) OR Dysuria OR Nocturia OR haematuria OR hematuria OR (Diarrhoea OR diarrhea OR Tenesmus OR (Rectal AND (pain OR bleed*)) OR Proctitis OR Incontinence OR Intestinal Toxicity) OR (“toxicit*” OR morbidity OR “side effect*” OR “complication*” OR “adverse effect*” OR “adverse event*” OR “symptom*”)) AND TI = (“radiotherap*” OR “radiation therap*” OR “stereotactic body*” OR “volumetric modulated arc therapy” OR “intensity modulated” OR “conformal radiotherapy” OR “3DCRT” OR “CRT” OR “SABR”) AND TI = (radiomic* OR feature* OR predict* OR model* OR correlat* OR corresp* OR depend* OR assoc* OR relat* OR interact* OR link* OR “risk factor*” OR analy*) AND TI = (prostate AND (cancer OR adenocarcinoma OR carcinoma))	573
Scopus	TITLE ((urinary AND frequen* OR urgen* OR retent* OR pain OR bleed* OR difficul* OR irritat* OR incontinence)) OR urethra* AND structure OR obstruct* )) OR dysuria OR nocturia OR haematuria OR hematuria OR (diarrhoea OR diarrhea OR tenesmus OR (rectal AND (pain OR bleed*)) OR proctitis OR incontinence OR intestinal AND toxicity) OR (“toxicit*” OR morbidity OR “side effect*” OR “complication*” OR “adverse effect*” OR “adverse event*” OR “symptom*”)) AND TITLE ((“radiotherap*” OR “radiation therap*” OR “stereotactic body*” OR “volumetric modulated arc therapy” OR “intensity modulated” OR “conformal radiotherapy” OR “3DCRT” OR “CRT” OR “SABR”) AND (radiomic* OR feature* OR predict* OR model* OR correlat* OR corresp* OR depend* OR assoc* OR relat* OR interact* OR link* OR “risk factor*” OR analy*) AND (prostate AND (cancer OR adenocarcinoma OR carcinoma)))	307
PubMed	((“Urinary Tract Symptoms”[Mesh] OR (“Urinary”[ti] AND (frequen*[ti] OR urgen*[ti] OR retent*[ti] OR pain[ti] OR bleed*[ti] OR difficul*[ti] OR irritat*[ti] OR incontinence[ti])) ) OR ( “Urethral Obstruction”[Mesh] OR (“Urethra*”[ti] AND (structure[ti] OR obstruct*[ti]))) OR Dysuria[Mesh] OR Dysuria[ti] OR Nocturia[Mesh] OR Nocturia[ti] OR Haematuria[Mesh] OR Haematuria[ti] OR Hematuria[Mesh] OR Hematuria[ti] OR ( (“Diarrhea”[Mesh] OR Diarrhea[ti]) OR Tenesmus[Mesh] OR Tenesmus[ti] OR (“Rectal Diseases”[Mesh] AND (“pain”[ti] OR “bleeding”[ti])) OR Proctitis[Mesh] OR Proctitis[ti] OR Incontinence[Mesh] OR Incontinence[ti] OR “Intestinal Toxicity”[ti]) OR ((“Toxicity”[Mesh] OR “toxicit*”[ti]) OR Morbidity[Mesh] OR Morbidity[ti] OR “Side Effects”[Mesh] OR “side effect*”[ti] OR “Complications”[Mesh] OR “complication*”[ti] OR “Adverse Effects”[Mesh] OR “adverse effect*”[ti] OR “adverse event*”[ti] OR Symptom*[ti])) AND (((“Radiotherapy”[Mesh] OR “radiotherap*”[ti]) OR “Radiation Therapy”[Mesh] OR “radiation therap*”[ti] OR “Stereotactic Body Radiotherapy”[Mesh] OR “stereotactic body*”[ti] OR “Volumetric Modulated Arc Therapy”[ti] OR “Intensity-Modulated Radiotherapy”[Mesh] OR “intensity modulated”[ti] OR “Conformal Radiotherapy”[Mesh] OR “conformal radiotherapy”[ti] OR “3DCRT”[ti] OR “CRT”[ti] OR “SABR”[ti]) AND ((“Radiomics”[Mesh] OR radiomic*[ti]) OR feature*[ti] OR predict*[ti] OR model*[ti] OR correlat*[ti] OR corresp*[ti] OR depend*[ti] OR assoc*[ti] OR relat*[ti] OR interact*[ti] OR link*[ti] OR “risk factor*”[ti] OR analy*[ti]) AND ((“Prostatic Neoplasms”[Mesh] OR (prostate[ti] AND (cancer[ti] OR adenocarcinoma[ti] OR carcinoma[ti])))))	708
